# Long non-coding RNA UCA1 promotes breast cancer by upregulating PTP1B expression via inhibiting miR-206

**DOI:** 10.1186/s12935-019-0958-z

**Published:** 2019-11-01

**Authors:** Yi Li, Qingan Zeng, Jiliang Qiu, Ting Pang, Jianzhong Xian, Xuexia Zhang

**Affiliations:** 1 Department of Thyroid & Breast Surgery, The Fifth Affiliated Hospital, Sun Yat-Sen University, Zhuhai, 519000 Guangdong Province People’s Republic of China; 2 Department of Surgery, Sun Yat-Sen University Cancer Center, State Key Laboratory of Oncology in South China, Collaborative Innovation Center for Cancer Medicine, Guangzhou, 510000 People’s Republic of China; 3 Department of Anesthesiology, The Sixth Affiliated Hospital, Sun Yat-Sen University, Guangzhou, 510655 Guangdong Province People’s Republic of China; 4 Department of Ultrasound, The Fifth Affiliated Hospital, Sun Yat-Sen University, Zhuhai, 519000 Guangdong Province People’s Republic of China; 5 Department of Anesthesiology, The Fifth Affiliated Hospital, Sun Yat-Sen University, No. 52 Meihua East Road, Xiangzhou District, Zhuhai, 519000 Guangdong Province People’s Republic of China

**Keywords:** lncRNA, UCA1, miR-206, PTP1B, Breast cancer

## Abstract

**Background:**

The long non-coding RNA (lncRNA) urothelial carcinoma-associated 1 (UCA1) is involved in various cancers and often functions through microRNAs. The pro-survival protein PTP1B is known to play important roles in cancer development. However, the connection between UCA1 and PTP1B in breast cancer is not well studied.

**Methods:**

In this study, we first evaluated the correlation between UCA1 level and PTP1B expression in breast tissues, which showed the expression of PTP1B were much higher in the breast tumor tissues than in the peritumor normal tissues. The UCA1 level was positively associated with PTP1B expression in breast tumor tissues.

**Results:**

We observed that UCA1 could up-regulate PTP1B expression in breast cancer cells. We also found that miR-206 could inhibit the expression of PTP1B by directly binding to the 3′-UTR of its mRNA. Interestingly, UCA1 could increase the expression of PTP1B through sequestering miR-206 at post-transcriptional level. The results also suggested that UCA1-induced PTP1B expression facilitated the proliferation of breast cancer cells.

**Conclusions:**

We conclude that UCA1 can up-regulates PTP1B to enhance cell proliferation through sequestering miR-206 in breast cancer. Our finding provides new insights into the mechanism of breast cancer regulation by UCA1, which could be a potential target for breast cancer treatment.

*Trial registration* 2012N5hSYSU48573. Registered at Oct 12, 2012

## Background

As the second most common cancer worldwide and the most frequent cancer in females, breast cancer is the leading cause of cancer-associated mortality among females and accounts for 23% of cancer caused death globally [1–3]. Although quite some advances have been achieved in its diagnosis and treatment, interventions are often not very effective because of the high proliferative ability of cancer cells and intrinsic resistance to clinical therapies [4]. Recent researches have shown that long non-coding RNAs (LncRNAs) have high potential as diagnosis and prognosis biomarkers and therapeutic targets in malignant tumors [5].

LncRNAs are > 200 nucleotides in length without protein-coding capacity that modulate several signaling pathways to serve oncogenic or tumor suppressive roles during tumorigenesis. LncRNAs can interact with macromolecules such as DNA, RNA or protein to exert cellar effects. Evidence has implicated that lncRNAs mainly developed cancer through epigenetic modulation, stimulation of oncogenic pathways and crosstalk with other RNA subtypes. In contrast, a novel lncRNAs were reported to have tumor suppressive effect in HCC suppress tumor growth [6, 7]. Urothelial cancer‐associated 1 (UCA1) is first identified as an oncogenic lncRNA in bladder cancer, which has been reported to regulate bladder cancer cell proliferation, migration, invasion chemoresistance, and metabolism [8]. Besides bladder cancer, oncogenic functions of lncRNA UCA1 were also identified in other cancers like breast cancer, colorectal cancer, esophageal squamous cell carcinoma, gastric cancer, hepatocellular carcinoma, melanoma, ovarian cancer, and tongue squamous cell carcinoma [9]. Besides the oncogenic function, lncRNA UCA1 was also found to regulate drug resistance in multiple types of malignant tumors [10]. For example, in breast cancer, UCA1 has been shown to induce drug resistance to tamoxifen in several recent studies [11–13]. UCA1 has been reported to bind to several miRNAs in different cancer cells, which include miR-193a in non-small cell lung cancer [14], miR-216b in hepatocellular cancer [15], miR-18a in breast cancer cells [16], miR-204 in colorectal cancer [17], etc.

miRNAs are small non-coding cellular RNAs which are ~ 22 nucleotides long and can repress their target genes by interfering with post-transcription pathways through cleaving mRNA molecules or inhibiting their translation [18]. In recent years, some miRNAs have been reported to be involved in cancer, playing important roles in many solid cancers, including breast cancer, pancreatic cancer, ovarian cancer and lung cancer [19, 20]. miR-206 was the first microRNA found in breast cancer, which plays an important role in cell apoptosis [21]. This microRNA is regarded as a suppressor in many other cancers [22, 23]. In breast cancer studies, a miR-206-binding site has been found within the 3′-untranslated regions (3′-UTR) of ER-α, and this microRNA is present at higher levels in MDA-MB-231 cells (ER-α negative) than in MCF-7 cells (ER-α positive) [24, 25]. In two most recent studies on human breast cancer, miR-206 was found to suppress Bcl-w expression [26] and FTH1P13 [27] by binding to the 3′-UTR regions in their mRNAs. Furthermore, miR-206 has been found to be connected with lncRNA UCA1. Yan et al. verified that knockdown of UCA1 could upregulate miR-206, which would suppress the growth of the cervical cancer cells.

Protein tyrosine phosphatase 1B (PTP1B) is a non-transmembrane protein tyrosine phosphatase that has been recognized as a critical regulator in various signaling pathways. PTP1B was initially identified as a tumor suppressor gene, but more recent studies have shown that PTP1B was commonly overexpressed in tumor tissues and it had a positive role in tumor development and progression, such as breast, prostate, colorectal, lung, and hepatocellular cancer [28–30]. PTP1B was well studied in breast cancer in the aspect of synergizing with the ErbB2 oncogene [31–33]. In a most recent study in gastric cancer it reported that PTP1B was inhibited by miR-338-3p via direct targeting to its 3′-UTR [34].

In this study, we aim to study whether lncRNA UCA1 promotes the development of breast cancer by targeting PTP1B through miR-206. Interestingly, we found that UCA1 could up-regulate PTP1B expression via sequestering miR-206 at post-transcription level, which led to the promotion of breast cancer. Our findings take a further step into the mechanism of lncRNA UCA1-mediated breast cancer growth.

## Methods and materials

### Patients and tissues

35 patients who were diagnosed with breast cancer were enrolled for this study. The proposed experimental plan and procedures have been reviewed and approved by the Ethics Committee of the the Fifth Affiliated Hospital, Sun Yat-Sen University (No. FAHSYSU201207223451). All 35 patients had signed the consent form to participate. Patient information was summarized in Additional file 1: Table S1. None of the patients had received radiotherapy or chemotherapy before surgery. Freshly frozen breast tumor tissues were obtained from these 35 patients, and noncancerous peritumor tissues were taken from the adjacent area of the tumor site used as control. All experiments were performed strictly in accordance with relevant guidelines and regulations.

### Cell lines

Two breast cancer cell lines, MCF-7 and MDA-MB-231, were used in this study. They were maintained in RPMI Medium 1640 (Invitrogen, USA) with 100 U/mL penicillin/streptomycin and 10% FBS.

### Plasmid construction

The full-length UCA1 and PTP1B were obtained from the cDNA of MCF-7 cells, which were cloned into pcDNA3.1 vector. The mutant of UCA1, without miR-206 binding site, was introduced into pcDNA3.1, termed as UCA1-mut. The information of primers and plasmid construction were listed in Additional file 1: Table S2. The fragment, which contained the miR-206 target site in the 3′-UTR of PTP1B mRNA, was cloned into pGL3-control vector to construct pGL-PTP1B-mut.

### Cell transfection

Cells were digested and made into cell suspension with the concentration of XX/Ml. And the XX mL cell suspension were seed in 6-well plates for overnight. Then they were transfected with plasmid or siRNAs by using Lipofectamine 2000 reagent (Invitrogen, Carlsbad, CA). All the transfections were proceeded according to the manufacturer’s protocol of Invitrogen. The siRNA fragments of UCA1 and PTP1B, miR-206 mimics, miR-206 inhibitor, and their respective negative controls were purchased from RiboBio (Guangzhou, China). And the detail information of these siRNA was summarized in Additional file 1: Table S2.

### Immunohistochemistry

Paraffin-embedded breast cancer tissue sections (4 µm) on poly-l-lysine-coated slides were deparaffinized and rinsed with 10 mmol/L Tris–HCl (pH 7.4) and 150 mmol/L sodium chloride. Peroxidase was quenched with methanol and 3% hydrogen peroxide. Slides were then placed in 10 mmol/L citrate buffer (pH 6.0) at 100 °C for 20 min in a pressurized heating chamber. After incubation with PTP1B primary antibodies (ab201974, Abcam, USA) for 1 h at room temperature, slides were thoroughly washed three times with phosphate-buffered saline. Bound antibodies were detected using the EnVision Detection Systems Peroxidase/DAB, Rabbit/Mouse Kit (Dako, Glostrup, Denmark). The slides were then counterstained with hematoxylin.

### Quantitative real-time PCR (qRT-PCR)

Total RNA from tissues or cultured cells was used to do reverse transcription by SuperScript™ IV Reverse Transcriptase (ThermoFisher Scientific, USA). To test miR-206 expression, poly (A) polymerase (Ambion, USA) was applied to polyadenylate total RNA. The qRT-PCR assay was applied using TransStart Top Green qPCR SuperMix (TransGen Biotech, China). The PCR reaction was evaluated using melting curve analysis. Quantitative PCR was conducted at 95 °C for 10 min followed by 40 cycles of 95 °C for 15 s and 60 °C for 60 s. Relative transcription alteration was evaluated as 2^−ΔΔCt^. GAPDH was used to normalize UCA1 and PTP1B. The level of miR-206 was normalized to U6 expression. Primers were shown in Additional file 1: Table S2.

### Western blot

Total cell lysates were prepared with sample buffer and boiled at 95 °C for 5 min. The samples were transferred to SDS–PAGE at 80 V for 3 h and then transferred to PVDF membranes for another 2 h. After incubation with specific antibodies for PTP1B (ab201974, Abcam, USA) or β-actin (ab8226, Abcam, USA) at 4 °C overnight, the membranes then were washed by 1% TBST for three times, incubated with secondary antibodies for 1 h and detected by chemiluminescence.

### Luciferase reporter gene assay

Cells were harvested and seeded with the concentration of 3 × 10^4^/well at 24-well plate. After growing at the plate for overnight, the cells were co-cultured with the pRL-TK plasmid containing the Renilla luciferase gene (Promega, Madison, WI) for internal normalization. Other plasmid containing PTP1B 3′-UTR, pCDNA3.1-UCA1, or their corresponding mutants were transfected into cells as the way of the pRL-TK plasmid. And then the luciferase activities were measured by the Dual-Luciferase reporter assay system (Promega, Madison, WI). All experiments were conducted at least three times.

### Cell proliferation assay

MCF-7 cells were plated at 1000 cells/well onto 96-well plates. After transfection cell proliferation was evaluated by 3-(4,5-dimethylthiazol-2-yl)-2,5 diphenyltetrazolium bromide (MTT) assays once per day for 3 days. Briefly, cells were cultured after different days (0, 1, 2, 3) and 15 μL of MTT was added to each well, followed by incubation for 4 h. The supernatant was discarded and 100 μL of dimethyl sulfoxide was added to stop the reaction. Absorbance at 490 nm was assessed using an ELISA reader system (Labsystem, Multiskan Ascent).

### BrdU incorporation assay

MCF-7 cells were seeded on 6-well plate and were grown overnight before transfection. Fresh medium containing 10 μmol/L BrdU (Sigma, USA) was used to incubate all groups for 4 h prior to immunofluorescence staining with mouse anti-BrdU antibody. After fixation with 4% paraformaldehyde in PBS, the cells were incubated overnight with a mouse anti-BrdU antibody (NeoMarkers, USA) and then treated with fluorescein isothiocyanate (FITC)-conjugated goat anti-mouse IgG (Dako, Glostrup, Denmark). DAPI was used to stain nuclei as the control to all cells in each group. The labeling index was expressed as the number of positively labeled nuclei/total number of nuclei.

### Colony formation assay

For colony formation analysis, treated cells at 1000 cells/well were seeded in 6-well plates with complete medium for 14 days. After methanol fixation and methylene blue staining, colonies were imaged and counted.

### In vivo tumorigenicity assay

BALB/C nude mice were purchased from Guangdong Medical Laboratory Animal Center, all of which were 4-week-old and male. And they were raised in specific-pathogen free environment and treated following the guidelines of the National Institutes of Health Guide for the Care and Use of Laboratory Animals. Also, all the experimental procedures would were approved by the Institute Research Ethics Committee at the Fifth Affiliated Hospital, Sun Yat-Sen University. Animal models were conducted as following steps. Firstly, MCF-7 cells were transfected with control, pCDNA3.1-UCA1, pCDNA- 3.1-UCA1 and si-PTP1B for 24 h. Then, these cells were harvested and re-suspended with sterile phosphate-buffered saline (PBS). The cell suspensions (0.2 mL) with the concentration of 2 × 10^7^ cell/mL were subcutaneously injected into the male BALB/c nude mice to build the tumor model. Each group had 6 nude mice. After a week from injection, the size of the tumors were measured by the length (L) and width (W). And the tumor volume (V) was calculated by with the formula (L × W2)/2. The mice were sacrificed and the tumors were excised and measured, after 42 days.

### Statistical analysis

Statistical analyses were carried out by using SPSS, version 19.0 (IBM Corp., Armonk, NY). All experiments were performed in triplicate. All data were presented in the form of mean ± standard deviation (SD). Student’s t-test or Wilcoxon’s signed-rank test, depending on data distribution, were used to compare mean values for independent groups to assess statistical significance. Pearson’s correlation coefficient was applied to analyze correlation between UCA1 level and PTP1B expression in breast cancer samples.

## Results

### PTP1B expression in breast tumor tissues is positively correlated to UCA1 level

**Fig. 1:**
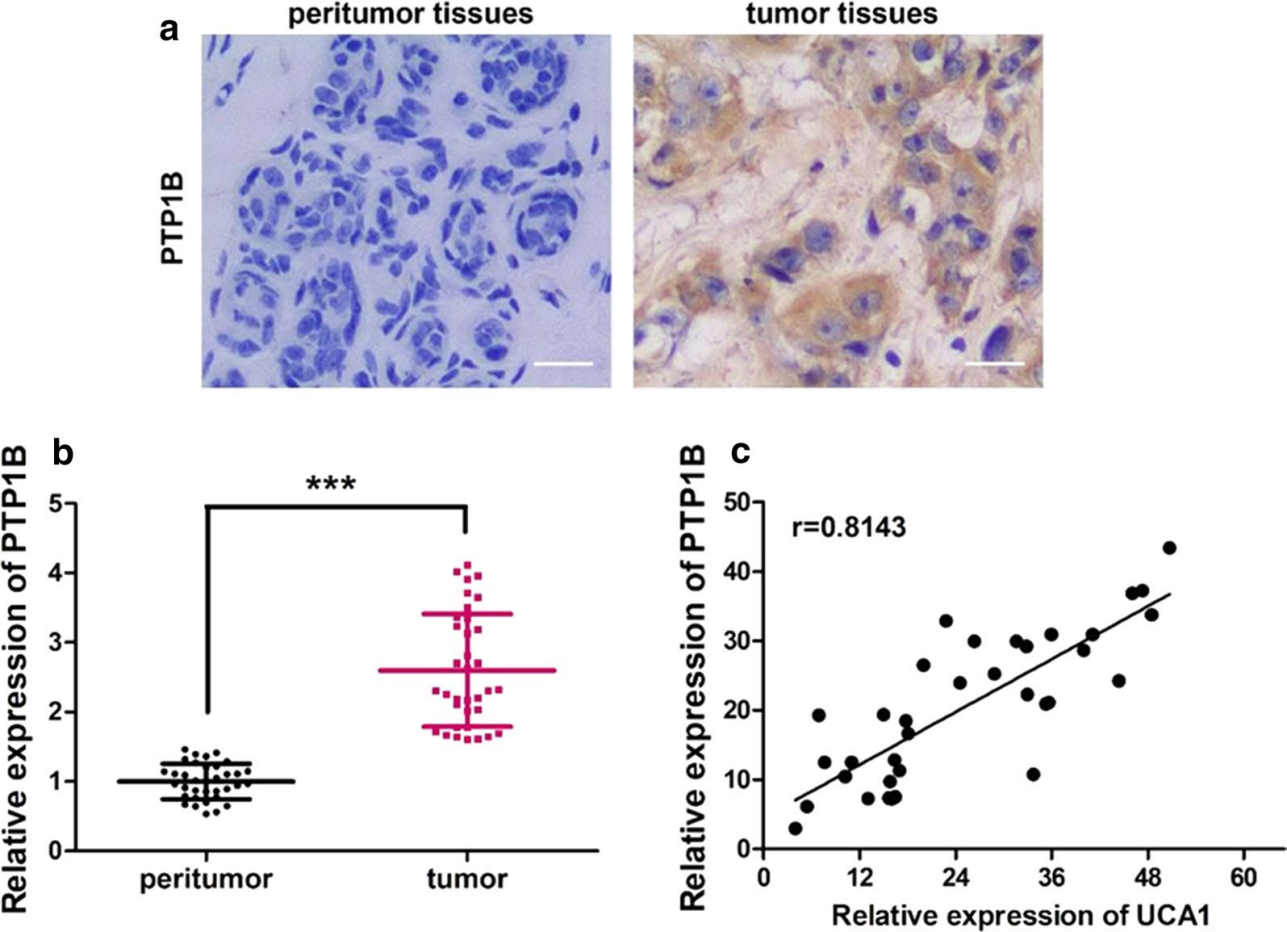
PTP1B is highly expressed in human breast tissues and is positively correlated to UCA1 level. **a** The expression of PTP1B examined by IHC staining in human breast tissues exhibited a much higher expression in the tumor tissues than the normal peritumor tissues. **b** PTP1B expression measured by qRT-PCR also showed a significant higher expression in the tumor tissues than the normal peritumor tissues assay in human breast cancer tissues and their paired noncancerous tissues (p < 0.001 by Wilcoxon’s signed-rank test). **c** Pearson’s correlation analysis on PTP1B expression and UCA1 level based on qRT-PCR data in breast tumor tissues suggested a strong positive correlation, with a correlation coefficient of 0.8143 (p < 0.001, n = 35)

PTP1B expression in breast cancer tumor tissue and peritumor tissue was examined by IHC staining. As shown in Fig. 1a, the color in the tumor issue after IHC staining is much darker than the peritumor tissue, which indicates that the expression of PTP1B in breast cancer tumor tissue is much higher than in the peritumor tissue.

This expression pattern was confirmed by the qRT-PCR experiment, which is presented in Fig. 1b. The qRT-PCR results were analyzed by Wilcoxon’s rank test, and PTP1B expression in tumor tissue is very significantly higher than in the peritumor tissue, whose p-value has a significant difference (p < 0.001).

The correlation between PTP1B expression in tumor tissue was analyzed by Pearson correlation method, which exhibited a strong positive correlation (Fig. 1c), which a correlation coefficient of 0.8143, and p value was less than 0.001.

### UCA1 up-regulates the expression of PTP1B in breast cancer cells

**Fig. 2:**
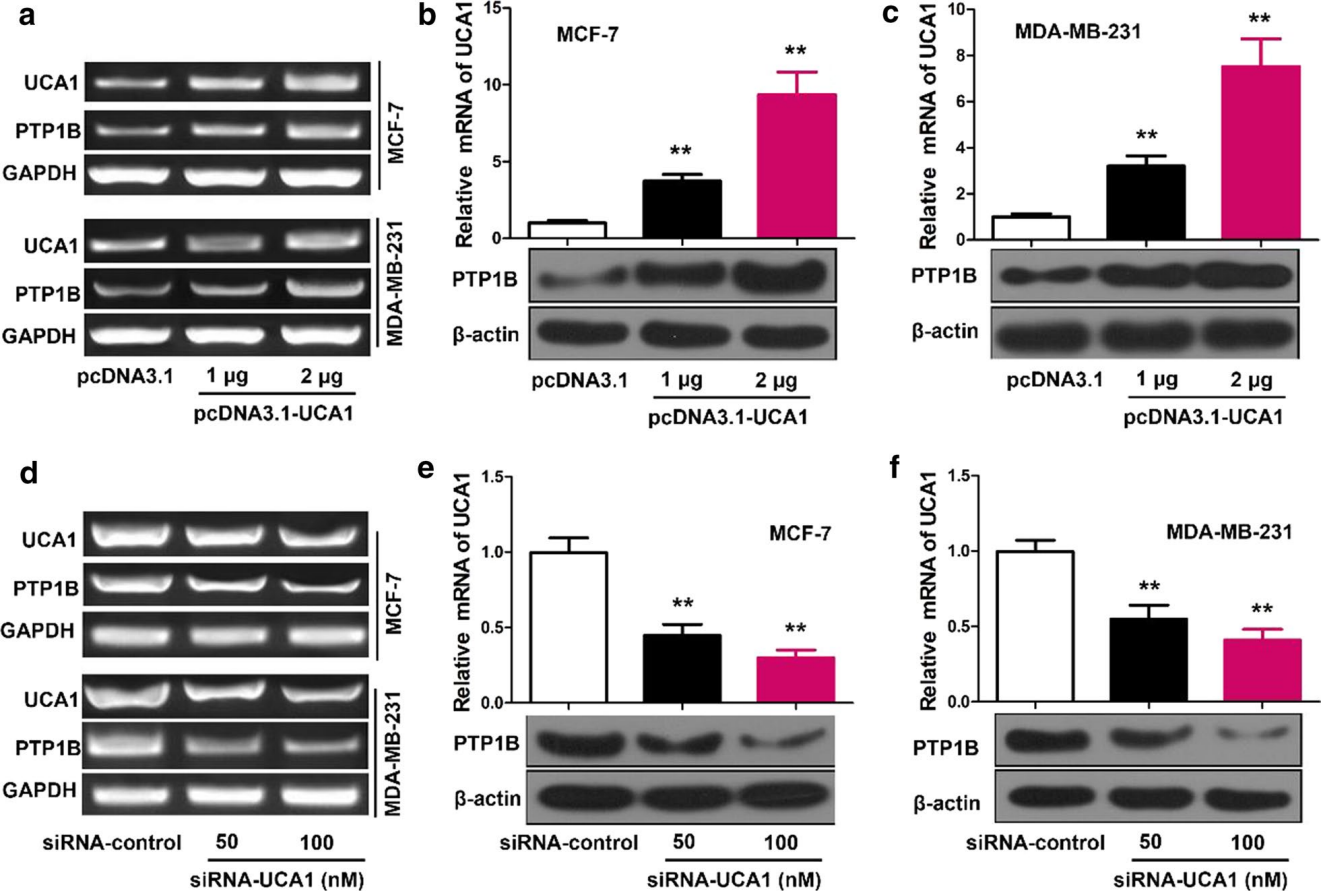
UCA1 up-regulates the expression of PTP1B in breast cancer cells. **a** MCF-7 and MDA-MB-231 cells were transfected with UCA1 or vector using Lipofectamine 2000 reagent. RNA was extracted 24 h after transfection. The mRNA levels of PTP1B was measured by RT-PCR analysis. **b**, **c** MCF-7 and MDA-MB-231 cells were transfected with UCA1 or vector using Lipofectamine 2000 reagent. Protein was extracted 48 h after transfection. The protein levels of PTP1B were examined by Western blot analysis. **d** MCF-7 and MDA-MB-231 were transfected with siRNA-UCA1 or siRNA-control using Lipofectamine 2000 reagent. RNA was extracted 24 h after transfection. The mRNA levels of PTP1B was tested by RT-PCR analysis. **e**, **f** MCF-7 and MDA-MB-231cells were transfected with siRNA-UCA1 or siRNA-control using Lipofectamine 2000 reagent. Protein was extracted 48 h after transfection. The protein levels of PTP1B was evaluated by Western blot analysis. Statistical significant differences are indicated: **p < 0.01, Student’s t-test

In both MCF-7 and MDA-MB-231 cells transfected with vector, 1 μg UCA1 or 2 μg UCA1, qRT-PCR showed that the mRNA levels of PTP1B increased when UCA1 levels increased (Fig. 2a). Western blot showed that the protein expression of PTP1B had significant increase in cells transfected with UCA1 than transfected with vector alone (p < 0.01), and in cells transfected with 2 μg UCA1 than in cells transfected with 1 μg UCA1 (p < 0.01), in both transfected MCF-7 (Fig. 2b) and MDA-MB-23 (Fig. 2c) cells.

Furthermore, when UCA1 was inhibited with specially synthesized siRNA, the mRNA levels of PTP1B decreased in cells transfected with siRNA-control, 50 nM siRNA, 100 nM siRNA (Fig. 2d), with the lowest in cells transfected with 100 nM siRNA. The protein expression of PTP1B were also significantly decreased in the order of cells transfected with siRNA-control, 50 nM siRNA, 100 nM siRNA, in both transfected MCF-7 (Fig. 2e) and MDA-MB-23 (Fig. 2f) cells (p < 0.01).

### MiR-206 suppresses PTP1B expression by targeting PTP1B mRNA 3′-UTR

**Fig. 3:**
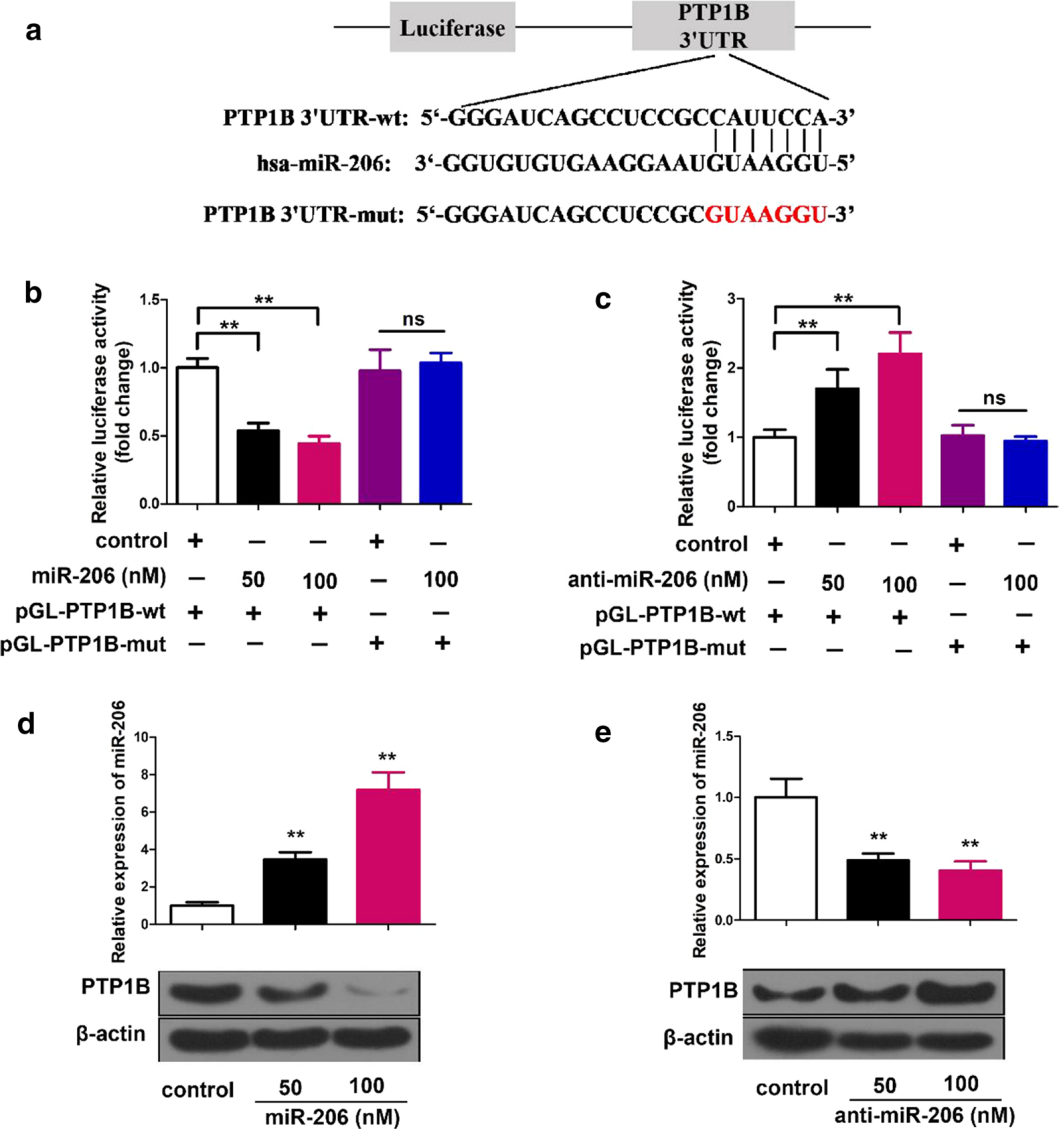
MiR-206 suppresses PTP1B expression via directly targeting PTP1B mRNA 3′UTR. **a** A model demonstrates the predicted conserved miR-206 binding site in the 3′UTR of PTP1B mRNA. Schematic diagram shows the miR-206 binding site-directed mutant of PTP1B mRNA 3′UTR. The PTP1B mRNA 3′UTR fragment containing wild type or mutant of miR-206-binding site was cloned into the downstream of the luciferase reporter gene in pGL3-control vector. **b**, **c** In MCF-7 cells regulation of miR-206 (or anti-miR-206) on pGL-PTP1B-wt and pGL-PTP1B-mut was tested by using luciferase reporter gene assay. **d**, **e** Effect of miR-206 (or anti-miR-206) on PTP1B expression was examined by Western blot. The level of miR-206 was analyzed by qRT-PCR assay after the cells were transfected with miR-206 or anti-miR-206. Statistical significant differences are indicated: **p < 0.01 and no significance (NS), Student’s t-test

Bioinformatics analysis showed that miR-206 shared complementary binding site with 3′-UTR of PTP1B mRNA (Fig. 3a). Luciferase reporter assay confirmed that the molecular binding of PTP1B mRNA and miR-206 (Fig. 3b, c). In MCF-7 cells transfected with 50 nM and 100 nM miR-206 with PTP1B wide type, relative luciferase activity was significantly decreased (p < 0.01), but when PTP1B was mutated at the putative miR-206 binding site the luciferase activity was completely restored, and the miR-206 at 100 nM had no difference to without it (Fig. 3b). In the luciferase report assays with anti-miR-206, anti-miR-206 could significantly increase the relative luciferase activity (p < 0.01). When the PTP1B mutant RNA replaced the wild type RNA, anti-miRNA-206 could not influence on the relative luciferase activity (Fig. 3c).

Suppression of PTP1B by miR-206 was also confirmed by western blot experiments (Fig. 3d, e). PTP1B expression was greatly reduced by infection with 50 nM miR-206, and much more greatly reduced by infection with 100 nM miR-206 (Fig. 3d; p < 0.01). On the other hand, when anti-miR-206 was used to replace miR-206, the PTP1B expression was greatly enhanced in cells transfected with 50 nM or 100 nM anti-miR-206 (Fig. 3e; p < 0.01).

### UCA1 elevates PTP1B expression through inhibiting miR-206

**Fig. 4:**
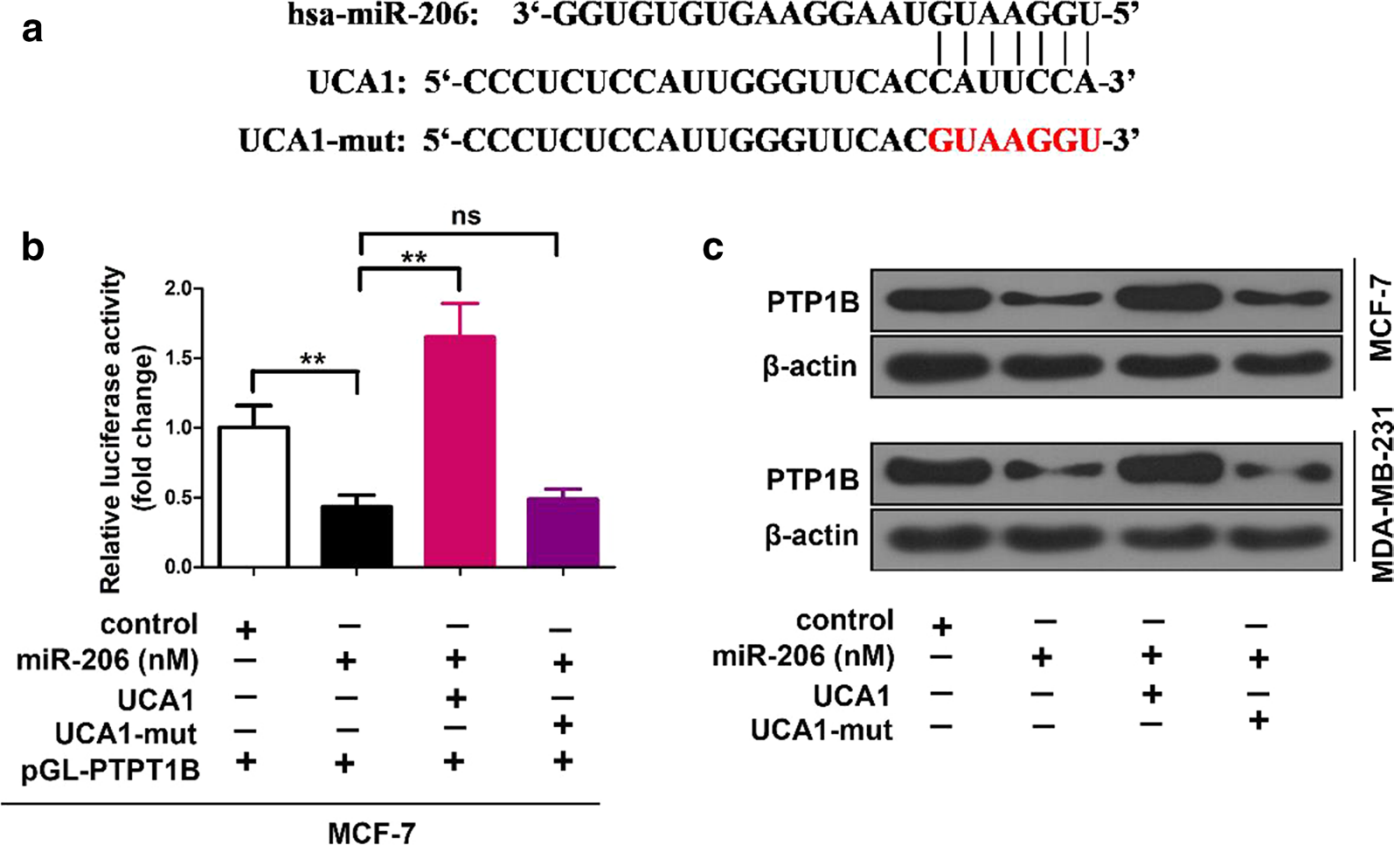
UCA1 is capable of elevating PTP1B expression through inhibiting miR-206. **a** Bioinformatics prediction of the interaction between UCA1 and miR-206 through complementary base-pairs was shown. The mutant sequence of UCA1 is indicated. **b** Luciferase activities of pGL-PTP1B were analyzed by luciferase reporter gene assay in miR-206 and/or UCA1 (or UCA1-206-mut) transfected MCF-7 cells. **c** In miR-206 and/or UCA1 (or UCA1-206-mut) treated cells, PTP1B expression was assessed by Western blot. **p < 0.01 and no significance (NS), Student’s t-test

Bioinformatics analysis also revealed that there is a putative miR-206 complementary binding site in UCA1 (Fig. 4a). In the luciferase reporter assay, miR-206 transfection led the relative luciferase activity to drop significantly (p < 0.01), and co-transfection with miR-206 and UCA1 greatly increased the relative luciferase activity (p < 0.01) (Fig. 4b). When UCA1 mutant RNA was used with miR-206 together in the transfection, the relative luciferase activity dropped to the same level as in the cells transfected only miR-206 (Fig. 4b).

In Western blot experiments, miR-206 greatly suppressed PTP1B expression, and adding UCA1 in transfection restored the PTP1B expression to the same level as without miR-206 (Fig. 4c). However, when UCA1 mutant was used to replace UCA1, the PTP1B expression were not restored and at the similar level to PTP1B expression in cells transfected with miR-206 (Fig. 4c).

### MiR-206/PTP1B signal mediates UCA1-accelerating cell proliferation in breast cancer

**Fig. 5:**
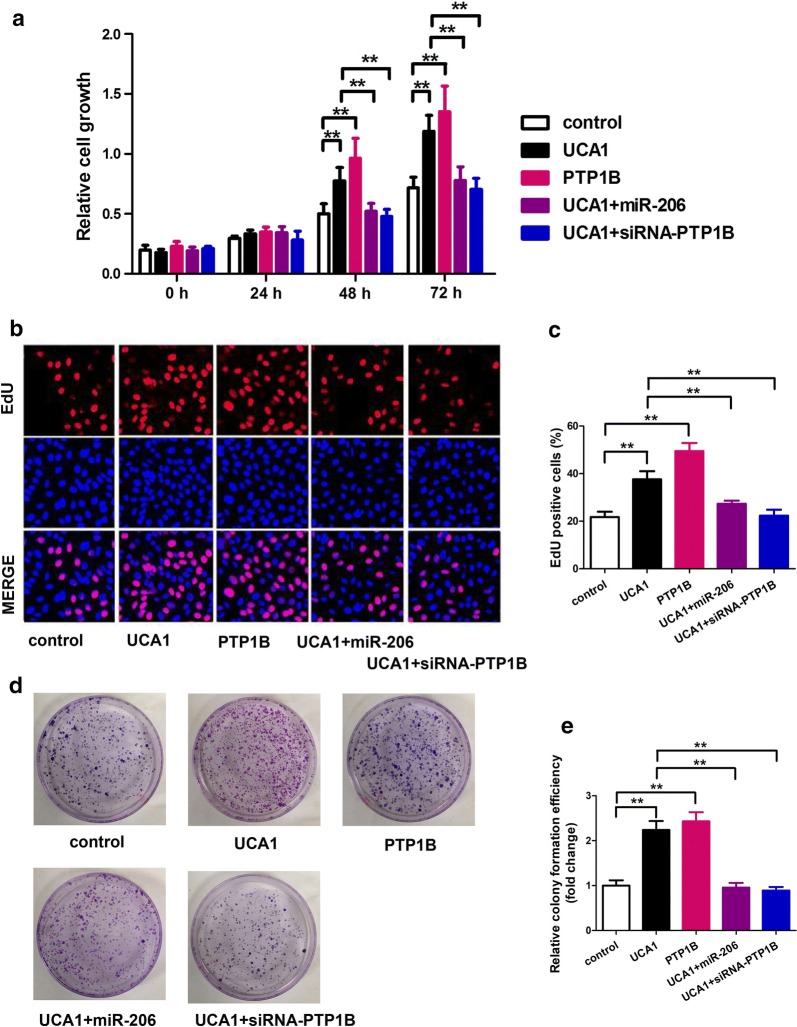
MiR-206/PTP1B signal mediates UCA1-accelerating cell proliferation in breast cancer. **a**–**c** Proliferation was tested by MTT and BrdU incorporation assays in MCF-7 cells treated with the indicated plasmids or siRNAs. **d**, **e** Colony formation of MCF-7 cells was counted post-transfection with the indicated plasmids or siRNAs

In the MTT assay, no significant differences were observed until 48 h post transfection. The two groups transfected with UCA1 or PTP1B had much higher relative cell growth compared to the control group (Fig. 5a; p < 0.01). The other two groups, which were transfected with UCA1 + miR-206 and UCA1 + siRNA-PTP1B respectively showed the same relative cell growth as the control group. At 72 h post transfection, the same patterns as at 48 h were observed.

Cell’s proliferation was also examined by BrdU incorporation assay. In the cells transfected with UCA1 or PTP1B, the EdU positive cells were significantly increased than the control group (Fig. 5b, c; p < 0.01). However, in the other two groups, which were transfected with UCA1 + miR-206 and UCA1 + siRNA-PTP1B respectively, showed similar EdU positive cells as the control group. There is no remarkable difference among control, UC1 + mir-206 and UCA1 + siRNA-PTP1B, which means UCA1 could regulate cell proliferation through functioning with mir-206.

This same phenomenon was also observed in colony formation experiment (Fig. 5d, e; p < 0.01). MCF-7 cells transfected with UCA1 or PTP1B formed much more colonies than the control group, but the other two groups transfected with UCA1 + miR-206 and UCA1 + siRNA-PTP1B respectively formed similar numbers of colonies as the control group (Fig. 5d). Figure 5e demonstrated the comparison of relative colony formation efficiency in fold change, which revealed the same observation. Moreover, the results also showed that the colony formation ability of MCF-7 cells infected with miR-206 were significantly down-regulated (Additional file 2: Figure S1, p < 0.01), which indicated that overexpressed miR-206 could inhibit the colony formation ability of MCF-7 cells.

### UCA1 promotes breast tumor cells growth through PTP1B in vivo

**Fig. 6:**
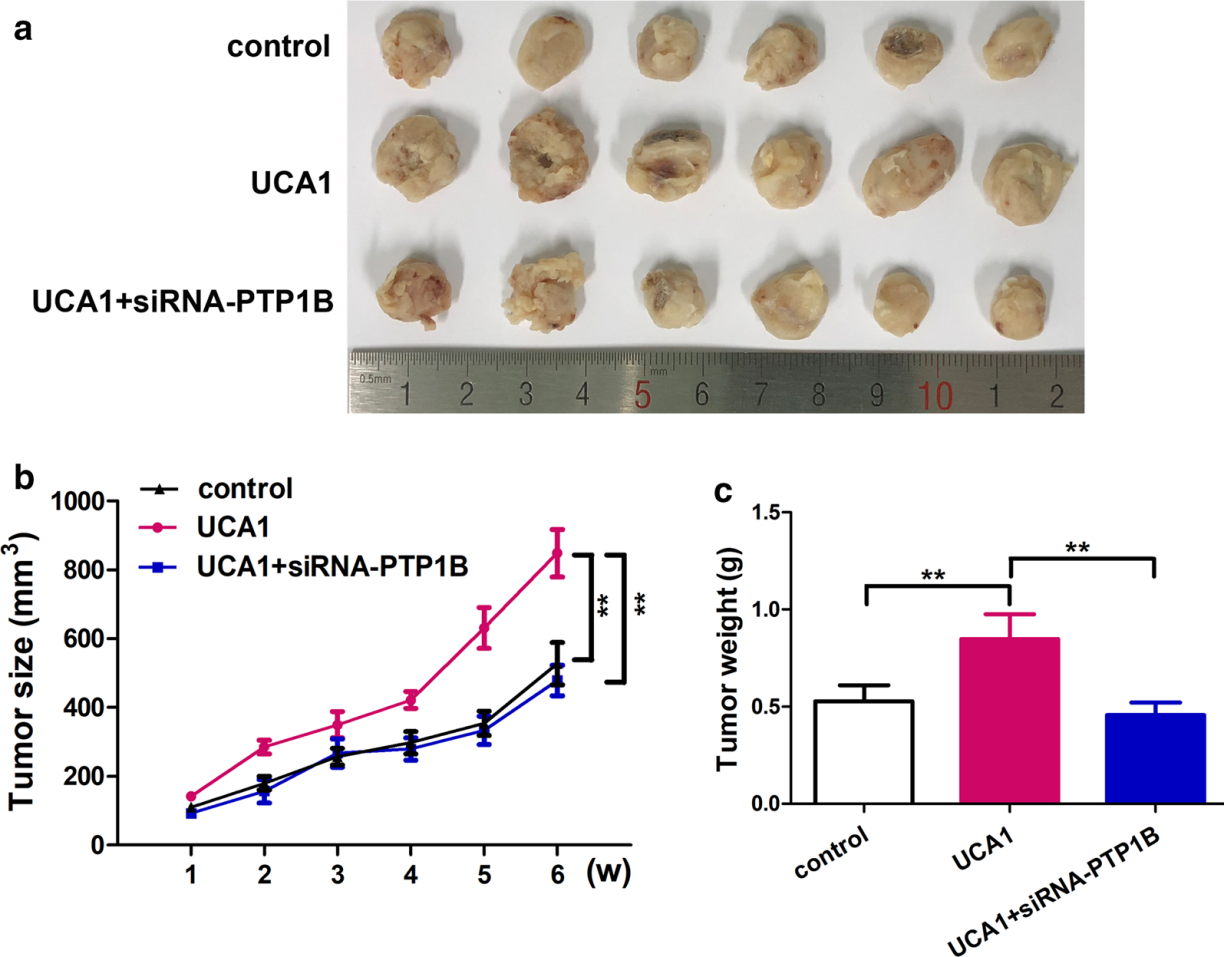
UCA1 promotes the growth of breast cancer cells through PTP1B in vivo. **a** The dissected tumors from nude mice were shown. **b** The growth curves of tumors from nude mice were shown. **c** The average tumor weight of each group was indicated

The in vivo tumorigenicity assay showed that UCA1 could promote the growth of breast tumor cells (Fig. 6). In Fig. 6a the dissected tumors from nude mice were presented to compare the difference of tumor sizes. The tumors from nude mice injected with UCA1 had the tumors in larger sizes than the control group. The tumors from nude mice injected with UCA1 and siRNA-PTP1B had similar size to the control group. The tumor size growth curve over 6 weeks clearly demonstrated that the tumors from nude mice injected with UCA1 grew much faster than the other groups, and the difference is significant (Fig. 6b; p < 0.01). The average tumor weights for each group were also compared, and the UCA1 group showed a larger average weight than the, other two groups (Fig. 6c; p < 0.01), which is in consistent with the tumor size and growth curve. The immunohistochemical images (Additional file 3: Figure S2) showed the protein expression of PTP1B was significantly increased in the group of UCA1, compared with control group. However, when co-transfect with UCA1 and siRNA-PTP1B, the protein expression of PTP1B decreased compared with the group of UCA1.

## Discussion

In this study we showed that PTP1B is highly expressed in breast tumor tissues and cells. This was also observed in other studies [33, 35]. In this study, we investigated the mechanism of PTP1B over expression in breast cancer, that UCA1 sequesters miR-206, so that miR-206 cannot inhibit PTP1B expression any more. PTP1B expression is positively corrected to UCA1 level in the breast cancer cells, both MCF-7 and MDA-MB-231 cell lines. MCF-7 and MDA-MB-231 cells were ER- positive and ER-negative breast cancer cells with low and high metastatic potential, respectively. This mean that the regulation of PTP1B might not be dependent on the expression of estrogen receptor (ER). This result was consistent with the previous results. For example, Soysal et al. [36] reported that PTP1B was an independent predictor of improved survival in breast cancer, and there was no association or functional impact of PTP1B expression in HER2(+) breast cancer. PTP1B up-regulation by UCA1 improved cell proliferation, and also promoted tumor cells growth. This is the first time to establish a connection between UCA1 and PTP1B expression in breast cancer, and also to reveal the mechanism of PTP1B expression up-regulation by UCA1 through sequestering miR-206. This gives new insights on UCA1 regulation in breast cancer.

**Fig. 7:**
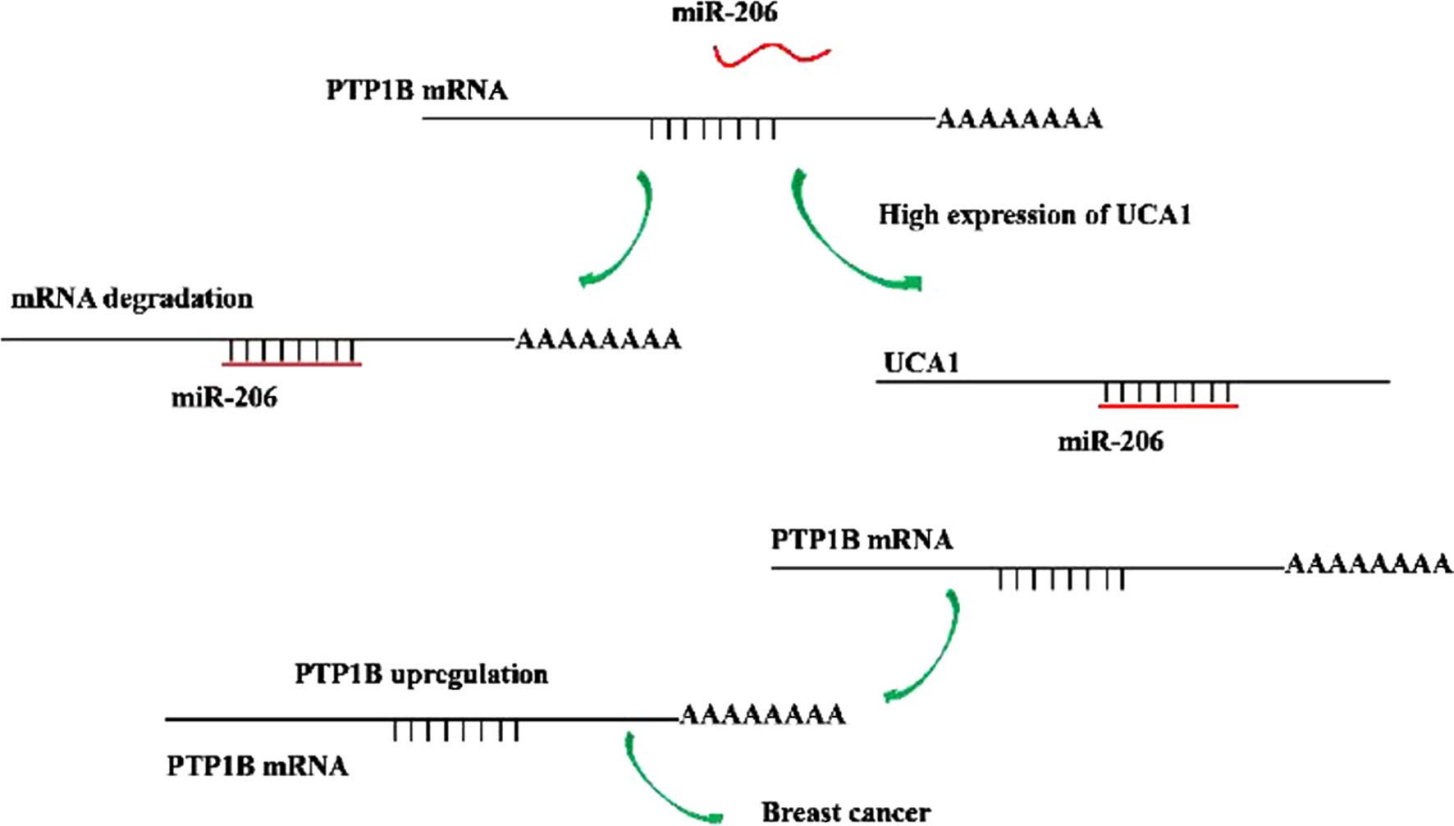
A model shows how UCA1 promotes breast cancer by up-regulating the expression of the PTP1B oncogene via sequestration of miR-206. Without the presence of UCA1, MiR-206 is capable of inhibiting the expression of PTP1B through directly binding to the 3′-UTR of PTP1B mRNA. When UCA1 is present, UCA1 can sponge miR-206, leading to the increase of PTP1B expression and results in the promotion of breast cancer growth

Based on what we observed in this study, we have proposed of putative model on how UCA1 up-regulates the expression of PTP1B and promotes breast cancer (Fig. 7). When there is no UCA1 present in the cells, miR-206 can suppress expression of PTP1B by directly binding to the 3′-UTR of its mRNA, which will lead to degradation of the mRNA and therefore inhibit the PTP1B expression. This will result in suppression of the tumor cells growth. When UCA1 is present in the cells, UCA1 can bind miR-206 at a more favorable affinity than PTP1B, and this will protect mRNA of PTP1B from miR-206, so PTP1B can be over expressed which will then promote the growth of breast cancer tumor cells. By this mechanism, UCA1 can up-regulate PTP1B expression and promote tumor development in breast cancer. UCA1 has also been shown to induce drug resistance to tamoxifen in breast cancer treatment [10, 11]. Our results may provide a possible explanation to the mechanism for the drug resistance induced by UCA1.

Similar mechanism involving miR-206 in breast cancer has also been observed in studies of other lncRNAs in cancel cells. For example, Ding et al. [26] reported that lncRNA HOTAIR could up-regulate Bcl-w expression to enhance cell proliferation through sequestering miR-206. Wang et al. [27] reported that lncRNA FTH1P3 could promote ABCB1 expression through targeting miR-206, which activates the chemo-resistance to paclitaxel, a first line chemotherapy drug in clinical oncology. It was also reported that lncRNA UCA1 acts as an endogenous sponge of miR-26a and downregulates miR-26a expression levels, and thereby relieving the inhibition of its target gene PTEN and alleviates VSMCs proliferation against atherosclerosis [32]. So, direct binding to microRNAs and then down-regulating these microRNAs’ levels, and relieving the inhibition of these microRNAs on their targets, may be a widely used mechanism that lncRNA employees to regulate signaling pathways in cells and cells proliferation.

In clinical oncology PTP1B has been highly validated as a therapeutic target for cancer [37], and extensive efforts have been made to develop active-site inhibitors for PTP1B [38]. Our results suggested some alternative ways to inhibit PTP1B expression, i.e. tumor suppression. One alternative is to mutate the miR-206 binding site on UCA1 to block the interaction between UCA1 and miR-206, which will have the same effect on inhibiting PTP1B expression. On the other hand, we can also mutate the UCA1 binding site on miR-206, so that UCA1 cannot sequester miR-206, and miR-206 can bind to PTP1B mRNA to inhibit its expression. However, this way may affect the possibility of down regulating PTP1B expression by miR-206, as the same site seems to bind both UCA1 and PTP1B. Further experiments are needed to further explore the feasibility of these two alternative ways. In our further research, aiming to deeply understand prognostic implications of these elements, the clinical assessment and the follow-up period of the patients as well as clinical implication of UCA1 and PTP1B expression will be studied.

## Conclusion

In breast tumor tissues PTP1B is highly expressed and the expression is positively correlated to UCA1 level. UCA1 can up-regulate the expression of PTP1B in breast cancer cells by sequestering miR-206 at post-transcriptional level, which can inhibit PTP1B expression by binding to the 3′UTR of its mRNA. We also demonstrated that up-regulation of PTP1B by UCA1 could enhance cell proliferation. Our finding provides new insights into the mechanism of breast cancer mediated by UCA1.

## Supplementary information

**Additional file 1: Table S1.** Clinical characteristics of patients enrolled in the study. **Table S2.** List of primers used in this study.

**Additional file 2: Figure S1.** Colony formation of MCF-7 cells was counted post-transfection with miR-206.

**Additional file 3: Figure S2.** The images of PTP1B in HCC tissue by immunohistochemistry assays confirm that UCA1 promotes the growth of breast cancer cells through PTP1B in vitro.

## Data Availability

The analyzed data sets generated during the study are available from the corresponding author on reasonable request.
