# Supplementary material for: Long non-coding RNA UCA1 promotes breast cancer by upregulating PTP1B expression via inhibiting miR-206
Source: Cancer Cell Int. 2019 Nov 1;19:275. doi: 10.1186/s12935-019-0958-z (PMC6824019; doi:10.1186/s12935-019-0958-z)
Supplement: Supplementary file 1 — Additional file 1: Table S1. Clinical characteristics of patients enrolled in the study. Table S2. List of primers used in this study. [file 12935_2019_958_MOESM1_ESM.docx]

**Additional Table S1**. Clinical characteristics of patients enrolled in the study

| **No.** | **Age** | **Sex** | **Organ** | **Pathology diagnosis** | **Grade** |
| --- | --- | --- | --- | --- | --- |
| 1 | 53 | F | Breast | Nonspecific infiltrating ductal carcinoma | I-II |
| 2 | 58 | F | Breast | Nonspecific infiltrating ductal carcinoma | II |
| 3 | 44 | F | Breast | Little nonspecific infiltrating ductal carcinoma | I |
| 4 | 68 | F | Breast | Little nonspecific infiltrating ductal carcinoma | I-II |
| 5 | 46 | F | Breast | Little nonspecific infiltrating ductal carcinoma | I |
| 6 | 63 | F | Breast | A little nonspecific infiltrating ductal carcinoma | I |
| 7 | 55 | F | Breast | Nonspecific infiltrating ductal carcinoma | I |
| 8 | 65 | F | Breast | Nonspecific infiltrating ductal carcinoma | I |
| 9 | 48 | F | Breast | Nonspecific infiltrating ductal carcinoma | II |
| 10 | 54 | F | Breast | Nonspecific infiltrating ductal carcinoma | I |
| 11 | 47 | F | Breast | Nonspecific infiltrating ductal carcinoma | I |
| 12 | 46 | F | Breast | Little nonspecific infiltrating ductal carcinoma | II |
| 13 | 48 | F | Breast | Nonspecific infiltrating ductal carcinoma | II |
| 14 | 34 | F | Breast | Nonspecific infiltrating ductal carcinoma | II |
| 15 | 41 | F | Breast | Nonspecific infiltrating ductal carcinoma | I |
| 16 | 33 | F | Breast | Nonspecific infiltrating ductal carcinoma | II-III |
| 17 | 52 | F | Breast | Nonspecific infiltrating ductal carcinoma | II |
| 18 | 39 | F | Breast | Nonspecific infiltrating ductal carcinoma | II |
| 19 | 43 | F | Breast | Nonspecific infiltrating ductal carcinoma | II |
| 20 | 46 | F | Breast | Nonspecific infiltrating ductal carcinoma | I |
| 21 | 44 | F | Breast | Nonspecific infiltrating ductal carcinoma | II |
| 22 | 61 | F | Breast | Nonspecific infiltrating ductal carcinoma | I-II |
| 23 | 48 | F | Breast | Nonspecific infiltrating ductal carcinoma | II |
| 24 | 60 | F | Breast | Nonspecific infiltrating ductal carcinoma | I-II |
| 25 | 46 | F | Breast | Nonspecific infiltrating ductal carcinoma | II |
| 26 | 41 | F | Breast | Nonspecific infiltrating ductal carcinoma | I |
| 27 | 63 | F | Breast | Nonspecific infiltrating ductal carcinoma | I |
| 28 | 50 | F | Breast | Little nonspecific infiltrating ductal carcinoma | II |
| 29 | 47 | F | Breast | Nonspecific infiltrating ductal carcinoma | I |
| 30 | 44 | F | Breast | Nonspecific infiltrating ductal carcinoma | II |
| 31 | 57 | F | Breast | Nonspecific infiltrating ductal carcinoma | I |
| 32 | 38 | F | Breast | Nonspecific infiltrating ductal carcinoma | I |
| 33 | 36 | F | Breast | Nonspecific infiltrating ductal carcinoma | II |
| 34 | 38 | F | Breast | Nonspecific infiltrating ductal carcinoma | II |
| 35 | 29 | F | Breast | Nonspecific infiltrating ductal carcinoma | I-II |

**Additional Table S2.** **List of primers used in this study**

**Primers for qRT-PCR**

| **Primer name 5**′**-3** | **Primer Sequences** |
| --- | --- |
| UCA1-F | TTTATGCTTGAGCCTTGA |
| UCA1-R | CTTGCCTGAAATACTTGC |
| PTP1B-F | TGGGTGAAGGAAGAGACCCA |
| PTP1B-R | CCCACGACCCGACTTCTAAC |
| GAPDH-F | ATCACCATCTTCCAGGAGCGA |
| GAPDH-R | CCTTCTCCATGGTGGTGAAGAC |
| miR-206-F | CCACACACTTCCTTACATTCCA |
| miR-206-R | GCGAGCACAGAATTAATACGAC |
| U6-F | CTCGCTTCGGCAGCACA |
| U6-R | AACGCTTCACGAATTTGCGT |

**Primers for subcloning and plasmid construction**

| **Primer name 5**′**-3** | **Primer Sequences** |
| --- | --- |
| pCDNA/UCA1-F | GGATCCGATCTCTCCTCTTCCTCCTG |
| pCDNA/UCA1-R | CTCGAGAGGAAGATTTCTTTTCTGTC |

**siRNA Oligos**

| **Primer name 5**′**-3** | **Primer Sequences** |
| --- | --- |
| SiRNA-UCA1-sense | TGGTAATGTATCATCGGCTTAGTTCAAGAGACTAAGCCGATGATACATTACCTTTTTTC |
| SiRNA-UCA1-antisense | TCGAGAAAAAAGGTAATGTATCATCGGCTTAGTCTCTTGAACTAAGCCGATGATACATTACCA |
| siRNA-PTP1B-sense | UGACCAUAGUCGGAUUAAATT |
| siRNA-PTP1B-antisense | UUUAAUCCGACUAUGGUCATT |
| siRNA-control-sense | TTTCTCCGAACGTGTCACGTTTCAAGAGAACGTGACACGTTCGGAGAATTTTTTC |
| siRNA-control-antisense | TCGAGAAAAAATTCTCCGAACGTGTCACGTTCTCTTGAAACGTGACACGTTCGGAGAAA |
