# Supplementary figures and images for: Long non-coding RNA UCA1 promotes breast cancer by upregulating PTP1B expression via inhibiting miR-206
Source: Cancer Cell Int. 2019 Nov 1;19:275. doi: 10.1186/s12935-019-0958-z (PMC6824019; doi:10.1186/s12935-019-0958-z)

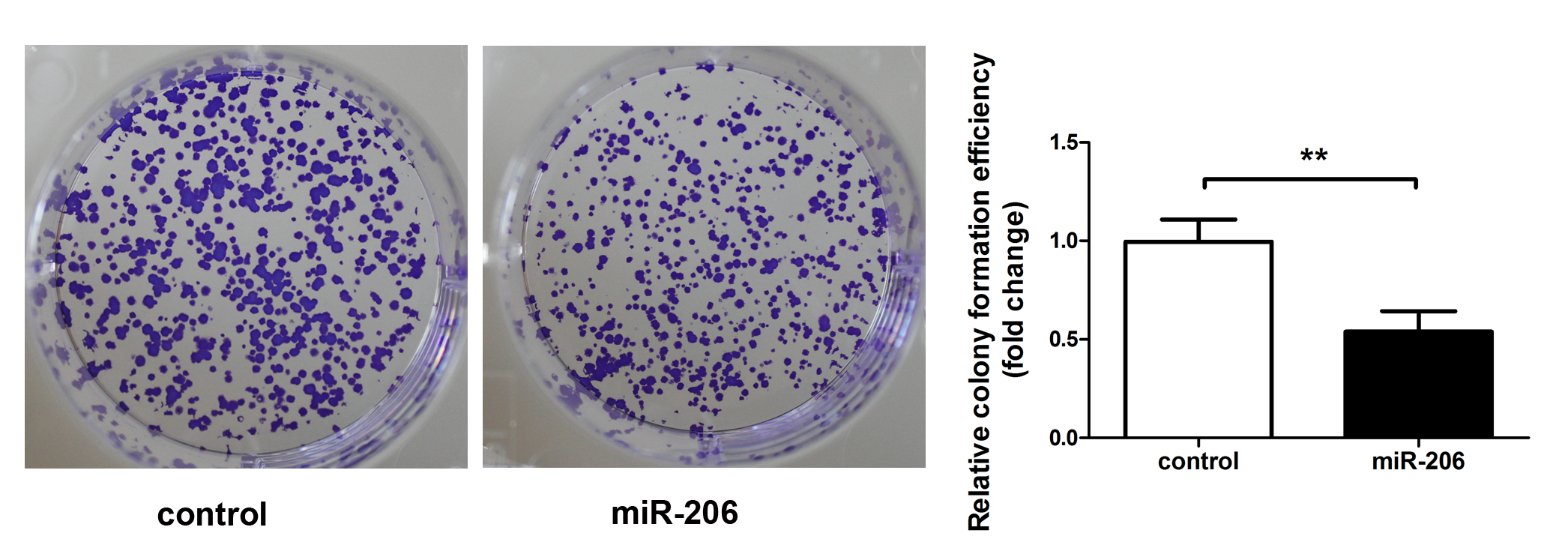

Supplement: Supplementary file 2 — Additional file 2: Figure S1. Colony formation of MCF-7 cells was counted post-transfection with miR-206. [file 12935_2019_958_MOESM2_ESM.tif]

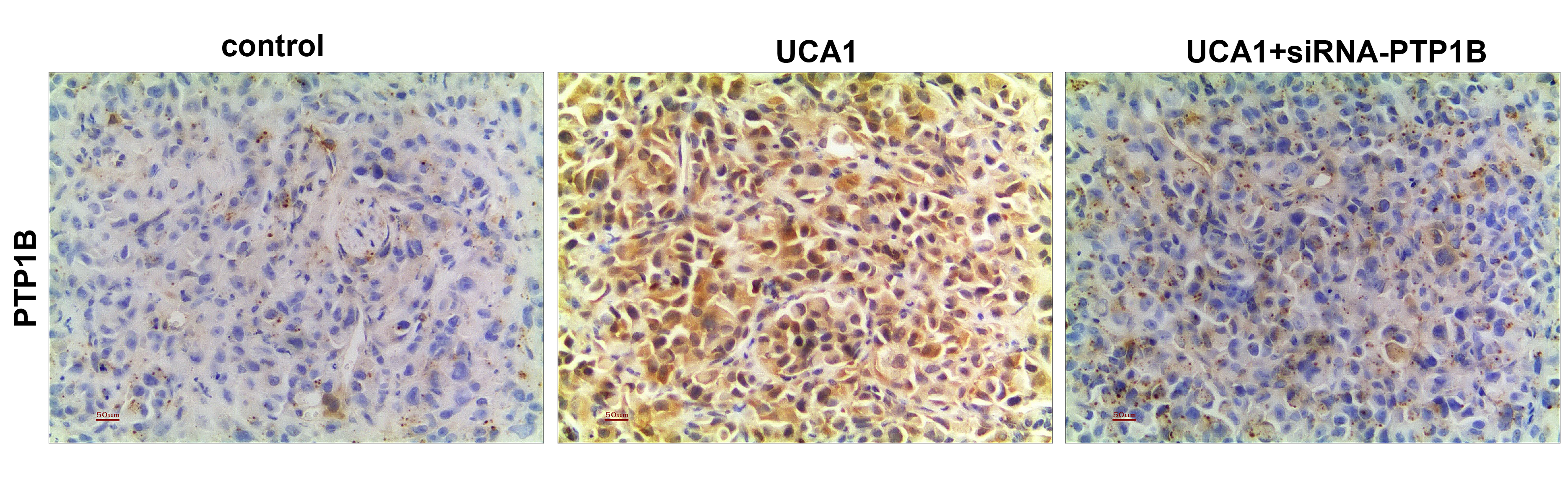

Supplement: Supplementary file 3 — Additional file 3: Figure S2. The images of PTP1B in HCC tissue by immunohistochemistry assays confirm that UCA1 promotes the growth of breast cancer cells through PTP1B in vitro. [file 12935_2019_958_MOESM3_ESM.tif]
